# Supplementary material for: Proteomic Studies of Primary Acute Myeloid Leukemia Cells Derived from Patients Before and during Disease-Stabilizing Treatment Based on All-Trans Retinoic Acid and Valproic Acid
Source: Cancers (Basel). 2021 Apr 29;13(9):2143. doi: 10.3390/cancers13092143 (PMC8125016; doi:10.3390/cancers13092143)
Supplement: Supplementary file 1 [file cancers-13-02143-s001.zip › cancers-1165153_supplementary/Supplementary figures and tables.pdf]

Supplementary Figures

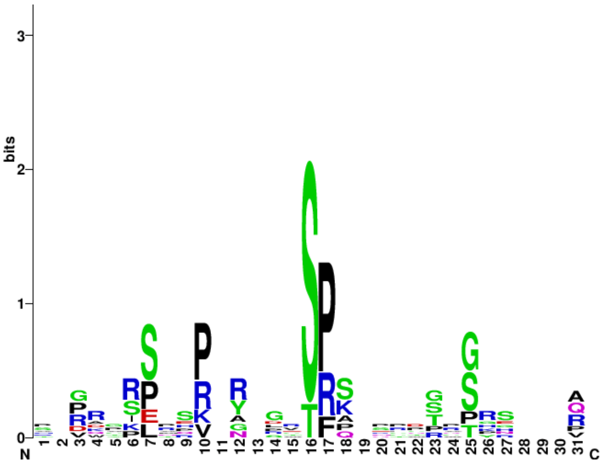

Figure S1. Sequence logo analysis of the 31 amino acid sequence windows surrounding the phosphorylation sites (located on position 16 on the x-axis) of the splicing factor network of Reactome pathway I showed in Figure 3c of the main text

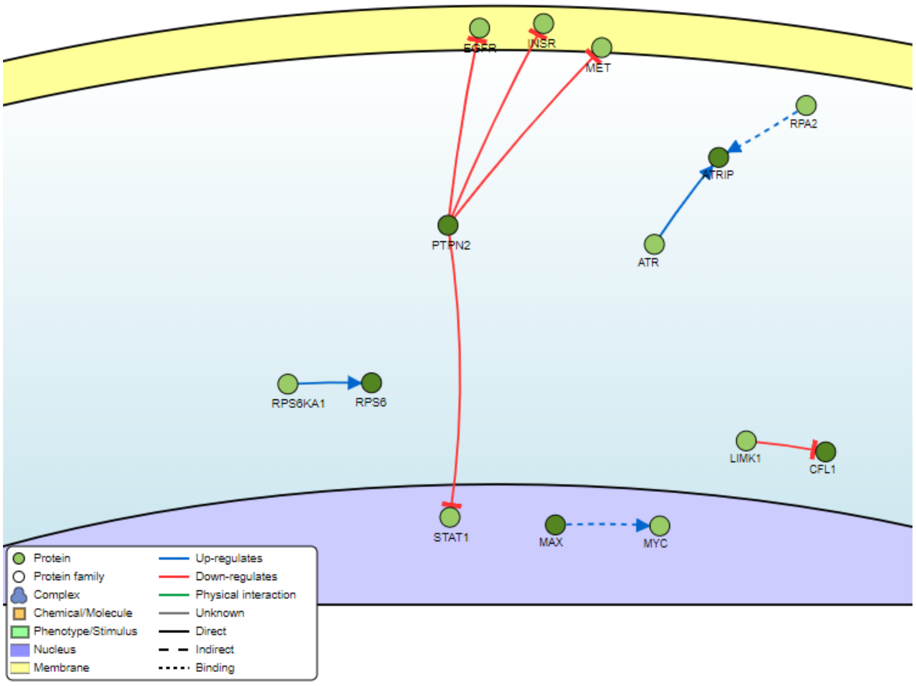

Figure S2. The analysis of the interactions of five differentially phosphorylated proteins from the PRE-R *vs* PRE-NR phosphoproteomic dataset (dark green circles) with signaling molecules from the SIGNOR database

Nodes and types of relationships are displayed as indicated at the bottom part of the design created by SIGNOR [53].

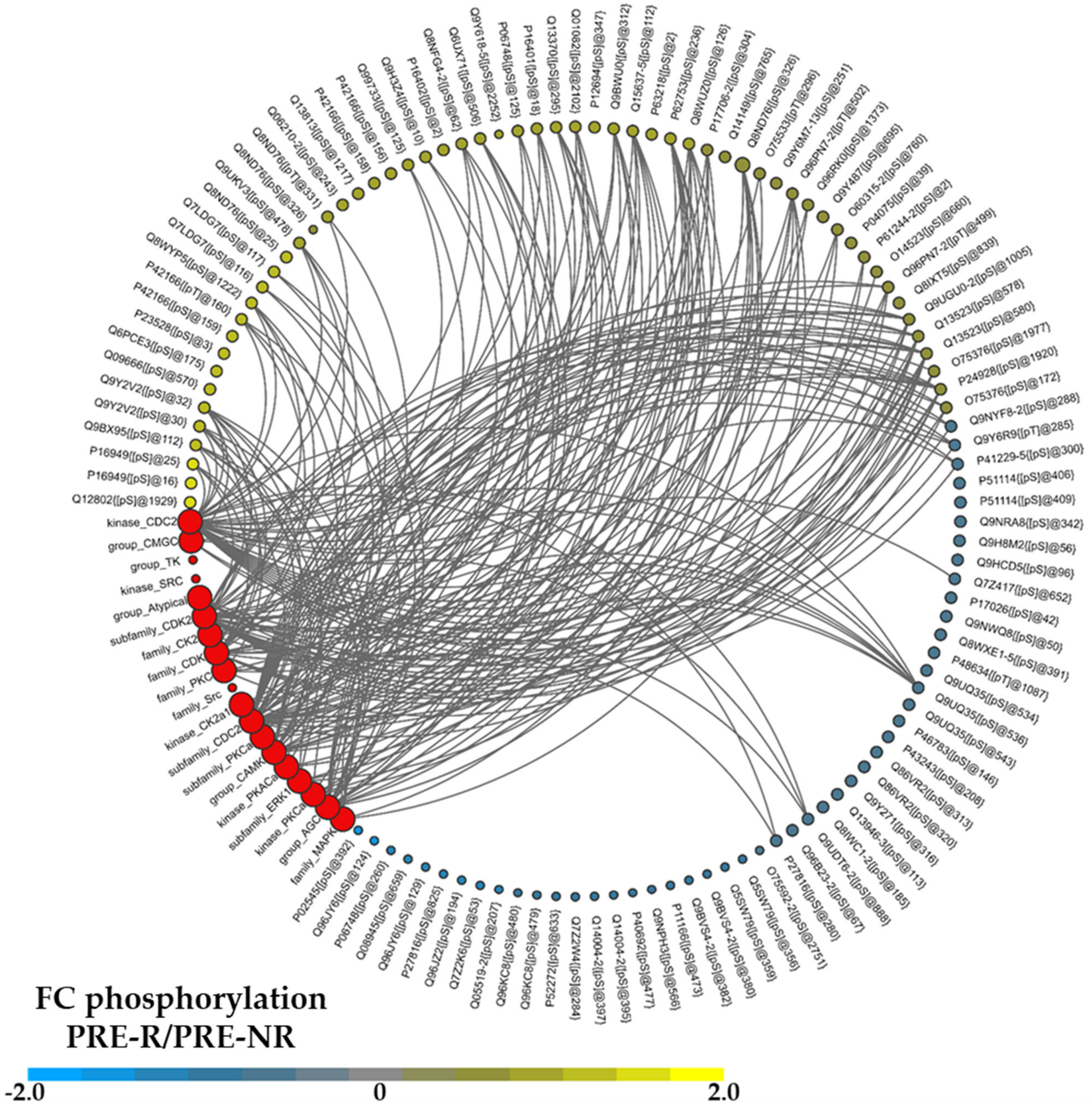

Figure S3. A circos layout linking the regulated PRE-R *vs* PRE-NR phosphoproteome with activated kinases (red nodes) predicted by the deep learning architecture DeepPhos performed in the piNET web platform

The change probability threshold was 0.9 [48]. Phosphosites nodes are colored according to their corresponding phosphorylation fold change (FC) as shown at the bottom of the plot [49].

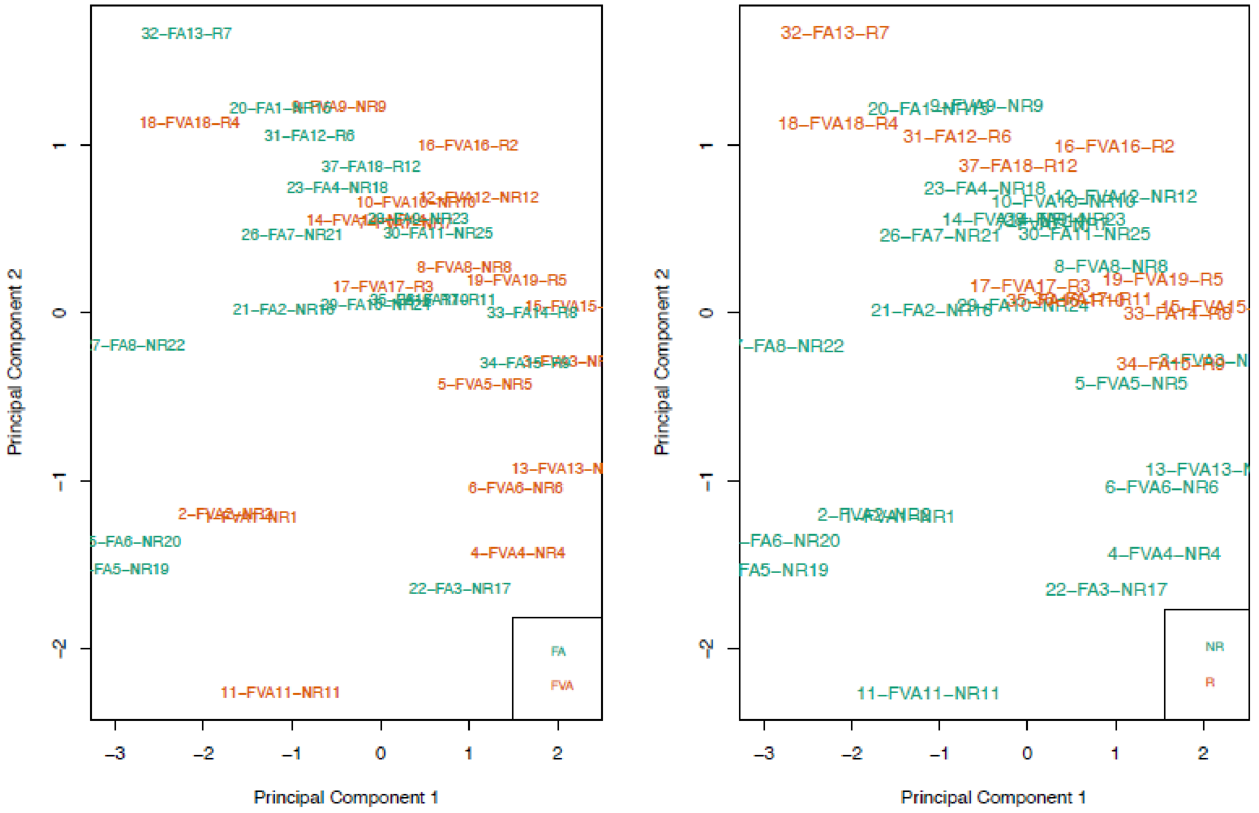

Figure S4. DNA methylation study of 12 PRE-R *vs* 25 PRE-NR patient samples before treatment

Principal component analysis plots of *M* values (*M* = log (Meth/Unmeth) calculated with the getM function from the minfi package). The top 1000 probes were used to calculate the pairwise distances to create the plots. The dataset was normalized with functional normalization and SNPs; cross-hybridizing probes and XY chromosome associated probes were filtered.

The left plot is colored by treatment (ATRA-VP-AraC, FVA in the plot annotations, in orange; ATRA-VP-TP, FA in the plot annotations, in green) while the right plot is colored by response (responders, R in the plot annotations, in orange; non-responders, NR in the plot annotations, in green). Fifteen out of the 25 PRE-NR and nine out of the 12 PRE-R patients were part of the MS-based proteomics and phosphoproteomics analyses presented in this study. The rest of the patients belonged to an external patient cohort.

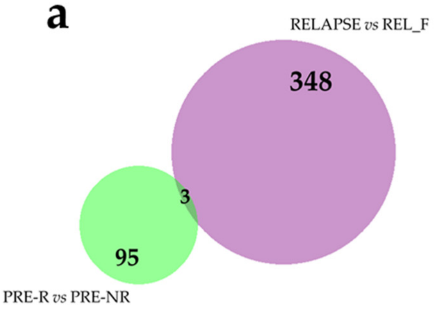

Gene	FC RELAPSE/REL_F	FC PRE-R/PRE-NR
PSTPIP1	-1.46	1.30
S100A8	-0.83	1.36
AGPAT5	1.00	-0.87

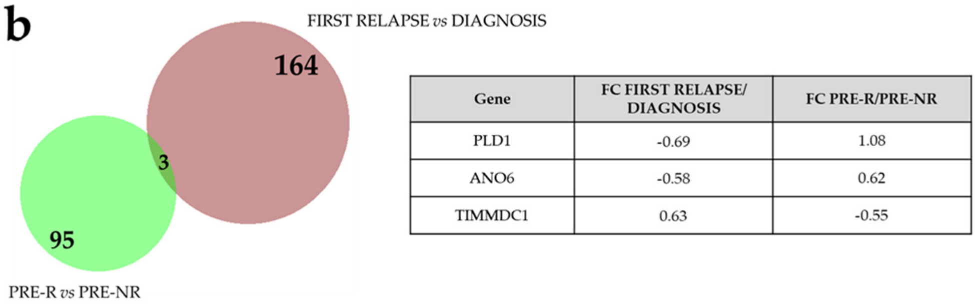

Gene	FC FIRST RELAPSE/ DIAGNOSIS	FC PRE-R/PRE-NR
PLD1	-0.69	1.08
ANO6	-0.58	0.62
TIMMDC1	0.63	-0.55

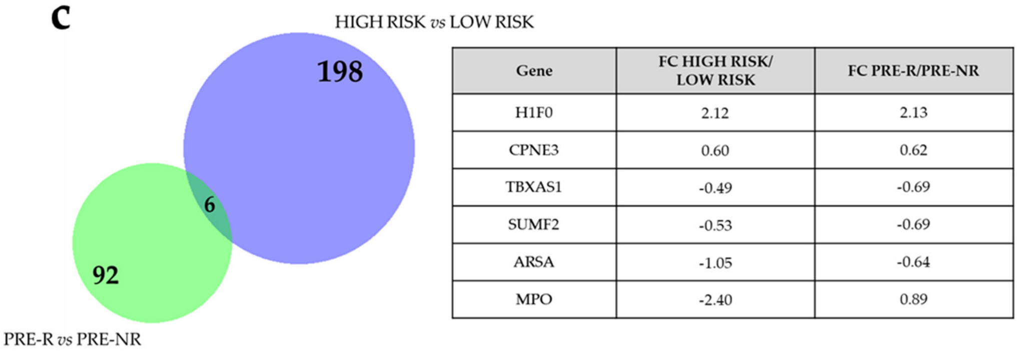

Gene	FC HIGH RISK/ LOW RISK	FC PRE-R/PRE-NR
H1F0	2.12	2.13
CPNE3	0.60	0.62
TBXAS1	-0.49	-0.69
SUMF2	-0.53	-0.69
ARSA	-1.05	-0.64
MPO	-2.40	0.89

Figure S5. Overlap of regulated proteins from the current PRE-R *vs* PRE-NR study with those identified in previously published MS-based proteomics studies

(a) Venn diagram of significantly regulated proteins identified in the current PRE-R *vs* PRE-NR and the published relapse *vs* relapse-free study [26]

(b) Venn diagram of significantly regulated proteins identified in the current PRE-R *vs* PRE-NR and the published first relapse *vs* first diagnosis study [71]

(c) Venn diagram of significantly regulated proteins identified in the current PRE-R *vs* PRE-NR and the published high-risk *vs* low-risk study [90]

(FC stands for expression fold change)

a

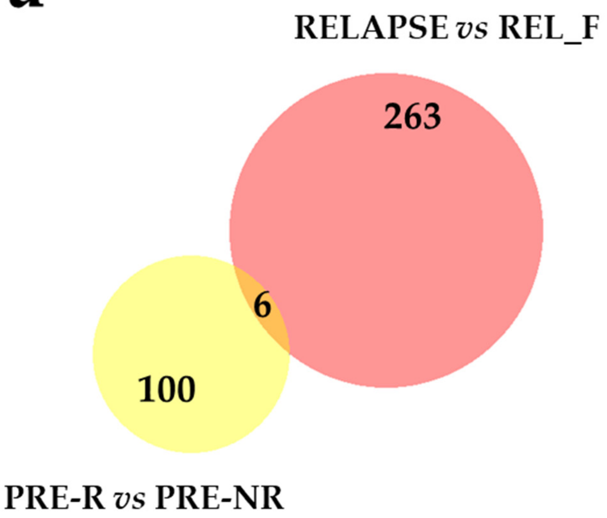

Phosphosite	FC RELAPSE/ REL_F	FC PRE-R/ PRE-NR
NPM1_S125	1.21	0.91
ZNF22_S42	0.85	-0.63
GFPT1_S243	-0.81	1.15
FAM134C_S320	0.62	-0.71
MAP7D3_S185	0.69	-0.76
DNAJC5_S10	-1.17	0.95

b

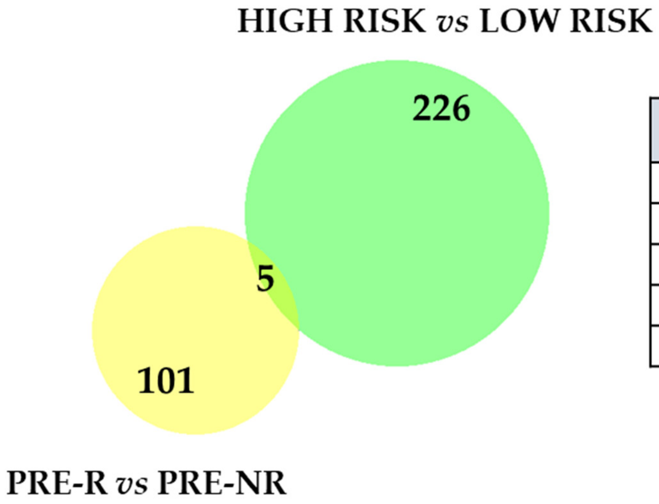

Phosphosite	FC HIGH RISK/ LOW RISK	FC PRE-R/ PRE-NR
STMN1_S16	0.74	1.61
ACIN1_S478	0.64	1.17
TMPO_T160	-0.86	1.25
TMPO_S156	-1.15	1.03
TMPO_S159	-1.17	1.25

Figure S6. Overlap of differentially regulated phosphorylation sites from the current PRE-R *vs* PRE-NR study with those identified in previously published MS-based phosphoproteomics studies

(a) Venn diagram of significantly regulated phosphorylation sites identified in the current PRE-R *vs* PRE-NR and the published relapse *vs* relapse-free study [26]

(b) Venn diagram of significantly regulated phosphorylation sites identified in the current PRE-R *vs* PRE-NR and the published high-risk *vs* low-risk study [90]

(None phosphosite was found overlapped between the current PRE-R *vs* PRE-NR and the published first relapse *vs* first diagnosis study [71])

(FC stands for phosphorylation fold change)

Supplementary Tables

Table S1. Proteomic differences between AML patients when comparing AML cells derived from responders *vs* non-responders to antileukemic treatment based on ATRA and VP

A description of differentially expressed proteins included in the neutrophil degranulation Reactome pathway of Figure 2c in the main text. The table presents the protein identity (gene name), a brief description of the protein functionality and keywords with regard to structure/function of the protein. The table is based on information from the Gene database and selected publications included in the PubMed database. Blue-colored cell indicates that the protein level was increased in non-responders; the other proteins showed increased levels in responders.

Protein (Gene ID)	Comment	Keywords
ANO6	**Anoctamin 6.** This encoded multi-pass transmembrane protein belongs to the anoctamin family. This protein is a part of a calcium-activated anion channel and is important for the calcium-dependent exposure of phosphatidylserine on the cell surface. The scrambling of phospholipid occurs in various biological systems, such as when blood platelets are activated, they expose phosphatidylserine. The protein is a regulator of sheddase activity, and its activity is regulated by the actin cytoskeleton. It also seems to be involved in regulation of apoptosis and cell growth, and these functions seem to be important in carcinogenesis. It can induce apoptosis through various mechanisms, and it is also involved in the regulation of exosomal release. The gene is expressed in many organs, including normal bone marrow and lymphoid organs [115–120]	Anion channel<br>Sheddase<br>Actin<br>Proapoptotic<br>Cancer
ARSA	**Arylsulfatase A**. Arylsulfatases are lysosomal enzymes that are important in cell metabolism. The encoded protein hydrolyzes cerebroside sulfate to cerebroside and sulfate. The gene is expressed in many organs, including normal bone marrow and lymphoid organs. Its expression is reduced in many malignancies, but the possible role of this decreased expression in carcinogenesis is not known [121]	Lysosome metabolism<br>Carcinogenesis?
CHI3L1	**Chitinase 3 like 1**. Chitinases catalyze the hydrolysis of the glycopolymer. The glycoside hydrolase 18 family of chitinases includes eight human family members. This gene encodes a glycoprotein member of the glycosyl hydrolase 18 family. The protein lacks chitinase activity and is secreted by activated macrophages, chondrocytes, neutrophils and synovial cells. It is thought to play a role in the process of inflammation and tissue remodeling, possibly through the stimulation of cytokine release and thereby modulation of the cancer cell microenvironment. The expression is high in normal bone marrow but is lower in lymph nodes [122]	Secreted protein<br>Myeloid cells<br>Cytokine induction<br>Carcinogenesis
CPNE3	**Copine 3**. Calcium-dependent membrane-binding proteins may regulate molecular events at the interface of the cell membrane and cytoplasm. This protein contains two type II C2 domains in the amino-terminus and an A domain-like sequence in the carboxy-terminus. The A domain mediates interactions between integrins and extracellular ligands. The gene is expressed in a wide range of organs including normal bone marrow, lymph nodes and spleen. High expression is associated with an adverse prognosis in human AML [123]	Adverse prognosis in AML<br>Cell membrane<br>Integrins

CSTB	**Cystatin B**. This protein functions as an intracellular thiol protease inhibitor. The protein is able to form a dimer stabilized by noncovalent forces, inhibiting papain and cathepsins l, h and b. The protein is thought to play a role in protecting against the proteases leaking from lysosomes. The expression is low in most organs, including normal bone marrow and lymphoid organs. Observations in an animal model of AML suggest that this molecule is increased during IL6-induced AML cell differentiation that finally leads to growth inhibition and apoptosis [124]	Lysosome<br>Myeloid differentiation<br>AML differentiation?
CTSG	**Cathepsin G**. This protein is a member of the peptidase S1 protein family and is found in azurophil granules (i.e., specialized lysosomes) of neutrophils. The protein participates in connective tissue remodeling. The gene shows high expression in normal bone marrow; the expression is very low or undetectable in other organs. The molecule is regarded as a member of the local bone marrow renin-angiotensin system that has been regarded as an important part of the bone marrow microenvironment, a regulatory system of normal bone marrow stem cells and possibly also a contributor in leukemogenesis [125]	Neutrophil<br>Myeloid differentiation<br>Stem cell niche<br>Peptidase<br>Leukemogenesis?
ELANE	**Elastase, neutrophil expressed**. Elastases form a subfamily of serine proteases that hydrolyze many proteins, including elastase and collagen-IV. The encoded preproprotein is proteolytically processed to generate the active protease. Following activation, this protease hydrolyzes proteins within neutrophil azurophil granules (specialized lysosomes), as well as proteins of the extracellular matrix. Mutations in this gene are associated with cyclic neutropenia and severe congenital neutropenia; some of these mutations also seem to be associated with an increased risk of AML/MDS [126]. The gene shows high expression in normal bone marrow, the expression is very low or undetectable in other organs	Neutrophil<br>Myeloid differentiation<br>Lysosome<br>Leukemogenesis<br>Protease<br>Elastin<br>Collagen
FGR	**FGR proto-oncogene, Src family tyrosine kinase**. This encoded protein is a member of the Src family of protein tyrosine kinases (PTKs). It is involved in mediating protein-protein interactions with phosphotyrosine-containing and proline-rich motifs. The protein localizes to plasma membrane ruffles, and functions as a negative regulator of cell migration and adhesion triggered by the beta-2 integrin signal transduction pathway. Normal bone marrow shows higher expression than other organs. This kinase supports AML proliferation and is associated with chemoresistance and it enhances growth factor dependent AML cell proliferation. High expression of the wild type protein seems to contribute to oncogenic transformation in human AML, and selective inhibition of this kinase suppresses *in vitro* and *in vivo* AML cell proliferation [127–129]	Tyrosine kinase<br>Integrin<br>Leukemogenesis
GCA	**Grancalcin**. This calcium-binding protein is abundant in neutrophils and macrophages. In the absence of divalent cation, this protein localizes to the cytosolic fraction; with magnesium alone, it partitions with the granule fraction; and in the presence of magnesium and calcium, it associates with both the granule and membrane fractions. The gene is expressed in many different organs/tissues, but high expression is seen especially in normal bone marrow. The protein can modulate intracellular signaling through interactions with Toll-like receptors, it can mediate chemoresistance in chronic myeloid leukemia cells through activation of autophagy, and it may influence autophagy as well as exocytosis through its interactions with annexin 7 [130–132]	Myeloid differentiation<br>Autophagy<br>Lysosome<br>Chemoresistance?
HLA-B	**Major histocompatibility complex, class I, B**. This HLA class I molecule is a heterodimer consisting of a heavy chain and a light chain (beta-2 microglobulin). The heavy chain is anchored in the membrane. Class I molecules play a central role in the immune system by presenting peptides derived from the endoplasmic	Immunoregulation<br>Antileukemic immune regulation?

	reticulum lumen. They are expressed in nearly all cells. The HLA class I molecules may also be important for antileukemic immunoregulation and thereby influence the prognosis in AML [133,134]	
LYZ	**Lysozyme**. This gene encodes human lysozyme. Lysozyme is one of the antimicrobial agents found in human milk, and is also present in spleen, lung, kidney, white blood cells, plasma, saliva, and tears. Lysozyme is present in neutrophil and monocyte granules, and it can also be expressed in AML cells [135]	Lysosome<br>AML
MCEMP1	**Mast cell expressed membrane protein 1**. This single-pass transmembrane protein is possibly involved in regulating mast cell differentiation. It shows high expression especially in normal bone marrow; its expression is lower in other organs. The molecule may also be expressed by other myeloid cells in certain biological contexts [136]	Mast cell differentiation?
MOSPD2	**Motile sperm domain containing 2**. The encoded protein is expressed by the endoplasmic reticulum and is important for the contact between endoplasmic reticulum and other organelles (e.g., endosomes, mitochondria, Golgi). It shows high expression in a wide range of tissues and is important for monocyte chemotaxis and activation [137–139]	Endoplasmic reticulum<br>Monocyte differentiation?
MPO	**Myeloperoxydase**. The myeloperoxidase (MPO) protein is synthesized during myeloid differentiation and constitutes the major component of neutrophil azurophilic granules. The mature myeloperoxidase is a tetramer composed of 2 light chains and 2 heavy chains. This enzyme is expressed only in bone marrow/myeloid cells, including most AML cells. The fraction of myeloperoxidase-positive AML cells varies between patients and is associated with distinct gene mutation patterns; favorable cytogenetic abnormalities are associated with high levels and for the intermediate AML risk group high expression seems to be associated with a favorable prognosis [140,141]	Myeloid differentiation<br>Favorable prognosis?
PLD1	**Phospholipase D1**. This gene encodes a phosphatidylcholine-specific phospholipase which catalyzes the hydrolysis of phosphatidylcholine in order to yield phosphatidic acid and choline. The enzyme may play a role in signal transduction and subcellular trafficking. Detectable expression is observed in several organs/tissues, including normal bone marrow. It localizes to secretory granules and lysosomes but can also be a regulator of p38 mediated signaling. The protein shows relatively high expression in human AML cells; this expression seems to be associated with differentiation, but low levels are also associated with an adverse prognosis [142–144]	Lysosome<br>Secretory granules<br>AML differentiation<br>AML prognosis
PRTN3	**Proteinase 3**. The gene shows high expression only in bone marrow with no detectable expression in lymphoid organs. This molecule is a regulator of normal hematopoietic stem cells, it is associated with myeloid differentiation in human AML and has a prognostic impact in several solid tumors (pancreatic, breast and renal cancer) [145–149]	Myeloid differentiation<br>Cancer prognosis<br>Hematopoietic stem cells
SERPINB1	**Serpin family B member 1**. The protein is a member of the serpin family of proteinase inhibitors. It inhibits the neutrophil-derived proteinases neutrophil elastase, cathepsin G, and proteinase-3 and thus protects tissues from damage at inflammatory sites [150]. Detectable expression in several organs, high expression especially in normal bone marrow	Inhibits neutrophil proteases

Table S2. Regulated proteins that have both phosphorylation and expression fold change (FC) in the PRE-R *vs* PRE-NR study

Gene	Protein name	Uniprot accession number	Amino acid	Position	Phosphorylation FC PRE-R/PRE-NR	Expression FC PRE-R/PRE-NR
CARHSP1	Calcium regulated heat stable protein 1	Q9Y2V2	S	30	1.56	0.92
CARHSP1	Calcium regulated heat stable protein 1	Q9Y2V2	S	32	1.56	0.92
DNAJC5	DnaJ heat shock protein family (Hsp40) member 5	Q9H3Z4	S	10	0.95	0.46
BCL7C	BAF chromatin remodeling complex subunit BCL7C	Q8WUZ0	S	126	0.80	0.51
MAP4	Microtubule associated protein 4	P27816	S	280	−0.79	−0.60
MAP4	Microtubule associated protein 4	P27816	S	825	−1.33	−0.60
PDLIM2	PDZ and LIM domain 2	Q96JY6	S	129	−1.42	−0.82
PDLIM2	PDZ and LIM domain 2	Q96JY6	S	124	−1.61	−0.82
					$R = 0.992$	

Table S3. Pair-wised fold change (FC) of regulated protein expression in responder patients considering quantitative values at pre-treatment (PRE), day 3 (3D) and day 8 (8D) of the ATRA-VP-TP treatment

PROTEOME OF RESPONDERS					
Uniprot accession number	Gene	Protein name	FC 3D/PRE	FC 8D/PRE	FC 8D/3D
Q9H019-3	*MTFR1L*	Mitochondrial fission regulator 1 like	0.58		−0.66
Q8IX01	*SUGP2*	SURP and G-patch domain-containing 2	0.53		
Q9BR77-2	*CCDC77*	Coiled-coil domain-containing 77	0.50		
P17026	*ZNF22*	Zinc finger protein 22	0.42		
Q00534	*CDK6*	Cyclin dependent kinase 6	0.39		
P55010	*EIF5*	Eukaryotic translation initiation factor 5	0.33		
P26373	*RPL13*	Ribosomal protein L13	0.33		
Q13144	*EIF2B5*	Eukaryotic translation initiation factor 2B subunit epsilon	0.32		
Q96AQ6	*PBXIP1*	PBX homeobox interacting protein 1	0.29		
P11234	*RALB*	Ras like proto-oncogene B	0.29		
P51608-2	*MECP2*	Methyl-CpG binding protein 2	0.28		
Q6UXN9	*WDR82*	WD repeat domain 82	−0.26	−0.39	
O75676-2	*RPS6KA4*	Ribosomal protein S6 kinase A4	−0.30		
Q9BYT8	*NLN*	Neurolysin	−0.33		
O00560-2	*SDCBP*	Syndecan binding protein	−0.39		
Q5MIZ7-3	*PPP4R3B*	Protein phosphatase 4 regulatory subunit 3B	−0.45		
Q8N335	*GPD1L*	Glycerol-3-phosphate dehydrogenase 1 like	−0.48		
P00915	*CA1*	Carbonic anhydrase 1		0.84	
P49588	*AARS1*	Alanyl-tRNA synthetase 1		0.74	
Q06203	*PPAT*	Phosphoribosyl pyrophosphatase amidotransferase		0.59	
P22732	*SLC2A5*	Solute carrier family 2 member 5		0.56	
Q9Y3A5	*SBDS*	SBDS ribosome maturation factor		0.56	
Q13526	*PIN1*	Peptidylprolyl cis/trans isomerase, NIMA-interacting 1		0.55	
O75312	*ZPR1*	ZPR1 zinc protein		0.51	0.80
Q8N0X7	*SPART*	Spartin		0.50	
P25815	*S100P*	S100 calcium binding protein P		0.49	
P48643	*CCT5*	Chaperonin containing TCP1 subunit 5		0.43	
Q6PGP7	*TTC37*	Tetratricopeptide repeat protein 37		0.42	
P22234	*PAICS*	; Phosphoribosylaminoimidazole carboxylase and phosphoribosylaminoimidazole-succinocarboxamide synthase		0.42	
P41250	*GARS1*	Glycyl-tRNA synthetase 1		0.38	
P46379-2	*BAG6*	BAG cochaperone 6		0.38	
Q13442	*PDAP1*	PDGFA associated protein 1		0.37	
O95139	*NDUFB6*	NADH:ubiquinone oxidoreductase subunit B6		−0.34	
Q93050-1	*ATP6V0A1*	ATPase H+ transporting V0 subunit a1		−0.35	
P61421	*ATP6V0D1*	ATPase H+ transporting V0 subunit d1		−0.35	
Q9HD20	*ATP13A1*	ATPase 13A1		−0.35	
Q9BSJ8	*ESYT1*	Extended synaptotagmin 1		−0.35	
Q96FV9	*THOC1*	THO complex 1		−0.35	
Q96DV4	*MRPL38*	Mitochondrial ribosomal protein L38		−0.35	

Q9NQ50	*MRPL40*	Mitochondrial ribosomal protein L4		−0.36	
O15269	*SPTLC1*	Serine palmitoyltransferase long chain base subunit 1		−0.36	
P07099	*EPHX1*	Epoxide hydrolase 1		−0.37	
Q86Y39	*NDUFA11*	NADH:ubiquinone oxidoreductase subunit A11		−0.38	
P11279	*LAMP1*	Lysosomal associated membrane protein 1		−0.38	−0.55
P30536	*TSPO*	Translocator protein		−0.39	
Q4KMQ2-3	*ANO6*	Anoctamin 6		−0.40	
Q9HD45	*TM9SF3*	Transmembrane 9 superfamily member 3		−0.40	
P83111	*LACTB*	Lactamase beta		−0.41	
P62873	*GNB1*	G protein subunit beta 1		−0.42	
Q7Z7H8	*MRPL10*	Mitochondrial ribosomal protein L10		−0.42	
P63218	*GNG5*	G protein subunit gamma 5		−0.42	
Q9BTD8-4	*RBM42*	RNA binding motif protein 42		−0.43	
Q9BWJ5	*SF3B5*	Splicing factor 3b subunit 5		−0.44	
Q9NVR2	*INTS10*	Integrator complex subunit 10		−0.46	
P0DOX5	*IGHG1*	Immunoglobulin heavy constant gamma 1		−0.46	
Q6IAN0	*DHRS7B*	Dehydrogenase/reductase 7B		−0.47	
P60468	*SEC61B*	SEC61 translocon subunit beta		−0.55	
Q96CU9-3	*FOXRED1*	FAD dependent oxidoreductase domain-containing 1		−0.57	
Q9H257	*CARD9*	Caspase recruitment domain family member 9		−0.57	
Q8NHQ9	*DDX55*	DEAD-box helicase 55		−0.61	
Q9NZK5	*ADA2*	Adenosine deaminase 2		−0.77	−0.57
P80723	*BASP1*	Brain abundant membrane attached signal protein 1			0.83
P06493	*CDK1*	Cyclin dependent kinase 1			0.81
Q06210-2	*GFPT1*	Glutamine--fructose-6-phosphate transaminase 1			0.81
Q9BTE3-2	*MCMBP*	Minichromosome maintenance complex-binding protein			0.69
P03915	*MT-ND5*	Mitochondrially enconded NADH:ubiquinone oxidoreductase core subunit 5			−0.57
Q9UDW1	*UQCR10*	Ubiquinol-cytochrome c reductase, complex III subunit X			−0.57
Q86WV6	*TMEM173*	Stimulator of interferon response cGAMP interactor 1			−0.57
P12694	*BCKDHA*	Branched chain keto acid dehydrogenase E1 subunit alpha			−0.58
P04439	*HLA-A*	Major histocompatibility complex, class I, A			−0.59
Q8IYJ3	*SYTL1*	Synaptotagmin like 1			−0.71
P13284	*IFI30*	IFI30 lysosomal thiol reductase			−1.36

Table S4. Pair-wised fold change (FC) of regulated protein expression in non-responder patients considering quantitative values at pre-treatment (PRE), day 3 (3D) and day 8 (8D) of the ATRA-VP-TP treatment

PROTEOME OF NON-RESPONDERS					
Uniprot accession number	Gene	Protein name	FC 3D/PRE	FC 8D/PRE	FC 8D/3D
O14519	*CDK2AP1*	Cyclin dependent kinase 2-associated protein 1	0.48		
Q96C01	*FAM136A*	Family sequence similarity 136 member A	0.41		
Q9NVC6	*MED17*	Mediator complex subunit 17	0.31	0.33	
P28799	*GRN*	Granulin precursor	0.31	0.31	
P40937	*RFC5*	Replication factor C subunit 5	0.31	0.27	
Q8ND56-2	*LSM14A*	LSM14 mRNA processing body assembly factor	0.25		
Q9NZK5	*CECR1*	Adenosine deaminase 2	−0.41		
P05109	*S100A8*	S100 calcium binding protein A8	−0.89		
P36222	*CHI3L1*	Chitinase 3 like 1		1.24	
P22732	*SLC2A5*	Solute carrier family 2 member 5		0.84	
P28907	*CD38*	CD38 molecule		0.74	
Q8IWZ8	*SUGP1*	SURP and G-patch domain-containing 1		0.66	
Q6P6C2	*ALKBH5*	Alkb homolog 5, RNA demethylase		0.63	
Q99543	*DNAJC2*	DnaJ heat shock protein family (Hsp40) member C2		0.49	
P21266	*GSTM3*	Glutathione S-transferase mu 3		0.47	
Q9BRJ6	*C7orf50*	Chromosome 7 open reading frame 50		0.44	
O14975	*SLC27A2*	Solute carrier family 27 member 2		0.44	
Q9UI36-2	*DACH1*	Dachshund family transcription factor 1		0.44	
Q9NX62	*IMPAD1*	3′(2′), 5′-biphosphate nucleotidase 2		0.43	
Q9UJX3	*ANAPC7*	Anaphase promoting complex subunit 7		0.42	
Q13085	*ACACA*	Acetyl-CoA carboxylase 1 alpha		0.41	
P33992	*MCM5*	Minichromosome maintenance complex component 5		0.39	
Q9Y5Q8-2	*GTF3C5*	General transcription factor IIIC subunit 5		0.39	
O43148	*RNMT*	RNA guanine-7 methyltransferase		0.37	
P33991	*MCM4*	Minichromosome maintenance complex component 4		0.37	
P49736	*MCM2*	Minichromosome maintenance complex component 2		0.37	
Q01826	*SATB1*	SATB homeobox 1		0.36	
P33993	*MCM7*	Minichromosome maintenance complex component 7		0.35	
Q7Z5L9-2	*IRF2BP2*	Interferon regulatory factor 2 binding protein 2		0.34	
P25205	*MCM3*	Minichromosome maintenance complex component 3		0.34	
Q9Y3Q3	*TMED3*	Transmembrane p24 trafficking protein 3		0.33	
Q14566	*MCM6*	Minichromosome maintenance complex component 6		0.32	
Q96P11-2	*NSUN5*	NOP2/Sun RNA methyltransferase 5		0.31	
Q15154	*PCM1*	Pericentriolar material 1		0.31	
Q9NRX2	*MRPL17*	Mitochondrial ribosomal protein L17		0.31	
P28340	*POLD1*	DNA polymerase delta 1, catalytic subunit		0.28	
P39748	*FEN1*	Flap structure-specific endonuclease 1		0.27	
Q15005	*SPCS2*	Signal peptidase complex subunit 2		0.27	
Q9UBU8-2	*MORF4L1*	Mortality factor 4 like 1		0.27	

Q6ZMZ3-2	*SYNE3*	Spectrin repeat containing nuclear envelope family member 3		−0.38	
Q9UNH7	*SNX6*	Sorting nexin 6		−0.39	
P13489	*RNH1*	Ribonuclease/angiogenin inhibitor 1		−0.40	
O43586	*PSTPIP1*	Proline-serine-threonine phosphatase-interacting protein 1		−0.40	
P84243	*H3F3A*	H3.3 histone A		−0.40	-0.39
Q13045	*FLII*	FLII actin remodeling protein		−0.41	
P49407-2	*ARRB1*	Arrestin beta 1		−0.42	
P0DMV9	*HSPA1B*	Heat schock protein family A (Hsp70) member 1B		−0.42	
P11215	*ITGAM*	Integrin subunit alpha M		−0.46	-0.44
P16070-18	*CD44*	CD44 molecule		−0.51	
Q13510	*ASAH1*	N-acylsphingosine amidohydrolase 1		−0.55	-0.34
O15145	*ARPC3*	Actin related protein 2/3 complex subunit 3		−0.55	
P42331-4	*ARHGAP25*	Rho GTPase activating protein 25		−0.55	
P61626	*LYZ*	Lysozyme		−0.56	
Q16644	*MAPKAPK3*	MAP kinase activated protein kinase 3		−0.58	
P12429	*ANXA3*	Annexin A3		−0.63	
P27707	*DCK*	Deoxycytidine kinase		−0.67	
O75351	*VPS4B*	Vacuolar protein sorting4 homolog B		−0.67	
Q7Z5R6	*APBB1IP*	Amyloid beta precursor protein binding family B member 1 interacting protein		−0.70	
O95466-2	*FMNL1*	Formin like 1		−0.73	
Q9BRF8	*CPPED1*	Calcineurin like phosphoesterase domain containing 1		−0.76	
Q12882	*DPYD*	Dihydropyrimidine dehydrogenase		−0.81	
P33241	*LSP1*	Lymphocyte specific protein 1		−1.11	
Q8N0X4	*CLYBL*	Cytramalyl-CoA lyase			0.40
Q9NYV4-2	*CDK12*	Cyclin dependent kinase 12			0.27
Q9BQ61	*TRIR*	Telomerase RNA component interacting RNase			−0.21
Q9C005	*DPY30*	Dpy-30 histone methyltransferase complex regulatory subunit			−0.22
Q9Y6M1	*IGF2BP2*	Insulin like growth factor 2 mRNA binding protein 2			−0.23
P63218	*GNG5*	G protein subunit gamma 5			−0.24
Q14254	*FLOT2*	Flotillin 2			−0.25
Q86Y39	*NDUFA11*	NADH:ubiquinone oxidoreductase subunit A11			−0.26
Q9UH99	*SUN2*	Sad1 and UNC84 domain containing 2			−0.28
Q14004	*CDK13*	Cyclin dependent kinase 13			−0.30
P05107	*ITGB2*	Integrin subunit beta 2			−0.31
Q15554	*TERF2*	Telomeric repeat binding factor 2			−0.31
P32942	*ICAM3*	Intercellular adhesion molecule 3			−0.31
Q8TBP6	*SLC25A40*	Solute carrier family 25 member 40			−0.46

Table S5. Pair-wised fold change (FC) of differentially regulated phosphorylation sites in responder patients considering quantitative values at pre-treatment (PRE), day 3 (3D) and day 8 (8D) of the ATRA-VP-TP treatment

PHOSPHOPROTEOME OF RESPONDERS					
Uniprot accession number	Phosphosite	Phosphoprotein name	FC 3D/PRE	FC 8D/PRE	FC 8D/3D
Q8WWM7	ATXN2L_S339	Ataxin 2 like	0.81		
Q16643	DBN1_S142	Drebrin 1	0.64		
Q8ND56-2	LSM14A_S192	LSM14 mRNA processing body assembly factor	−0.37		0.54
Q641Q2-2	WASHC2A_S284	WASH complex subunit 2A	−0.42		
Q9H501	ESF1_S198	ESF1 nucleolar pre-rRNA processing protein homolog	−0.43		
O15164-2	TRIM24_S777	Tripartite motif containing 24	−0.44		
P62753	RPS6_S240	Ribosomal protein S6	−0.44		
Q86WB0	ZC3HC1_S344	Zinc finger C3HC-type containing 1	−0.45		
Q92576-2	PHF3_S1837	PHD finger protein 3	−0.53		
Q9NQC3-5	RTN4_S184	Reticulon 4	−0.53		
Q9UH99	SUN2_S12	Sad1 and UNC84 domain containing 2	−0.54		
Q9Y580	RBM7_S204	RNA binding motif protein 7	−0.57	−0.44	
Q9UKV3	ACIN1_S240	Apoptotic chromatin condensation inducer in the nucleus	−0.77		
Q9UKV3	ACIN1_S243	Apoptotic chromatin condensation inducer 1	−0.77		
O43598	DNPH1_S12	2'-deoxynucleoside 5'-phosphate N-hydrolase 1		1.14	
Q13459	MYO9B_S1267	Myosin-IXB		0.93	
Q13459	MYO9B_T1271	Myosin-IXB		0.93	
Q13459	MYO9B_S1290	Myosin-IXB		0.93	
O14683	TP53I11_S14	Tumor protein p53 inducible protein 11		0.74	
Q15424-4	SAFB_S601	Scaffold attachment factor B		0.69	
Q15424-4	SAFB_S604	Scaffold attachment factor B		0.69	
P14317	HCLS1_S275	Hematopoietic cell-specific Lyn substrate 1		0.69	
P23588	EIF4B_S406	Eukaryotic translation initiation factor 4B		0.68	
P23588	EIF4B_S409	Eukaryotic translation initiation factor 4B		0.68	
Q6UN15	FIP1L1_S500	Factor interacting with PAPOLA and CPSF1		0.64	
Q7Z2W4	ZC3HAV1_S275	Zinc finger CCCH-type containing, antiviral 1		0.58	
P48634	PRRC2A_S1089	Proline rich coiled-coil 2A		0.56	
P23588	EIF4B_S406	Eukaryotic translation initiation factor 4B		0.56	
Q9UMZ2-5	SYNRG_S1075	Synergin gamma		0.55	
Q9Y520-4	PRRC2C_S878	Proline rich coiled-coil 2A		0.54	
Q86TB9-4	PATL1_S177	PAT1 homolog 1, processing body mRNA decay factor		0.50	
Q66K74-2	MAP1S_S631	Microtubule associated protein 1S		0.48	
Q6Y7W6	GIGYF2_S236	GRB10 interacting GYF protein 2		0.46	
Q04637-8	EIF4G1_S1210	Eukaryotic translation initiation factor 4 gamma 1		0.46	
P48634	PRRC2A_S1085	Proline rich coiled-coil 2A		0.46	
Q92538-3	GBF1_S1318	Golgi brefeldin Aresistant guanine nucleotide exchange factor 1		0.46	

Q92597	NDRG1_S330	N-myc downstream regulated 1		0.44	
O75427	LRCH4_S513	Leucine rich repeats and calponin homology domain containing 4		0.44	
Q9GZY6	LAT2_S44	Linker for activation of Tcells family member 2		−0.39	
Q9Y618-3	NCOR2_S2049	Nuclear receptor corepressor 2		−0.40	
Q9Y618-3	NCOR2_S2057	Nuclear receptor corepressor 2		−0.40	
O75494-2	SRSF10_S133	Serine andarginine rich splicing factor 10		−0.43	
Q8NDT2	RBM15B_S609	RNA binding motif protein 15B		−0.47	
Q9P1Y6	PHRF1_S1124	PHD and ring finger domains 1		−0.58	
Q9H1E3	NUCKS1_S214	Nuclear casein kinase and cyclin dependent kinase substrate 1		−0.61	
Q53EL6-2	PDCD4_S302	Programmed cell death 4		−0.62	
P05114	HMGN1_S7	High mobility group nucleosome binding domain 1		−0.64	−0.42
Q8TC07-2	TBC1D15_S70	TBC1 domain family member 15		−0.72	
O75533	SF3B1_T257	Splicing factor 3b subunit 1		−0.79	
Q09666	AHNAK_S2397	AHNAK protein		−0.94	
P42166	TMPO_S159	Thymopoietin		−1.63	
P42166	TMPO_T160	Thymopoietin		−1.63	
Q9Y3Z3	SAMHD1_S33	SAM and HD domain containing deoxynucleoside triphosphate			0.52
Q8WWQ0	PHIP_S674	Pleckstrin homology domain interacting protein			−0.46
O75592-2	MYCBP2_S3467	MYC binding protein 2			−0.52

Table S6. Pair-wised fold change of differentially regulated phosphorylation sites in non-responder patients considering quantitative values at pre-treatment (PRE), day 3 (3D) and day 8 (8D) of the ATRA-VP-TP treatment

PHOSPHOPROTEOME OF NON-RESPONDERS					
Uniprot accession number	Phosphosite	Phosphoprotein name	FC 3D/PRE	FC 8D/PRE	FC 8D/3D
P20700	LMNB1_S391	Lamin B1	1.22		
P10412	HIST1H1E_T18	H1.4 linker histone, cluster member	0.63		
Q8IY67-2	RAVER1_S617	Ribonucleoprotein, PTB binding 1	0.48		
Q8IYB3	SRRM1_S743	Serine andarginine repetitive matrix 1	0.41		
Q9ULU4-22	ZMYND8_S510	Zinc finger MYND-type containing 8	0.40		
Q9NYF8-2	BCLAF1_S383	BCL2 associated transcription factor 1	0.38		
Q9NYF8-2	BCLAF1_S387	BCL2 associated transcription factor 1	0.38		
P43243	MATR3_S604	Matrin 3	0.35		
Q96KR1	ZFR_S1054	Zinc finger RNA binding protein	0.35		
Q9NYV4-2	CDK12_T1244	Cyclin dependent kinase 12	0.35		
Q9GZY8-2	MFF_S131	Mitochondrial fission factor	−0.37	−0.37	
Q5T1M5	FKBP15_S1164	FKBP propyl isomerase family member 15	−0.38		
P06702	S100A9_T113	S100 calcium binding protein A9	−0.47		
Q14204	DYNC1H1_S4368	Dynein cytoplasmic 1 heavy chain 1	−0.51		
P16070-18	CD44_S304	CD44 molecule	−0.51		
Q05655	PRKCD_S645	Protein kinase C delta	−0.54		
Q09666	AHNAK_S93	AHNAK nucleoprotein	−0.63		
Q12802	AKAP13_S2563	A-kinase anchor protein 13	−0.81		
B2RUZ4	SMIM1_S22	Small integral membrane protein 1	−0.83	−0.67	
Q9BVC5	C2orf49_S189	Chromosome 2 open reading frame 49		0.96	
Q9BVC5	C2orf49_S193	Chromosome 2 open reading frame 49		0.96	
Q9NXE4-2	SMPD4_S246	Sphingomyelin phosphodiesterase 4		0.71	
Q9C0C2	TNKS1BP1_S1666	Tankyrase 1 binding protein 1		0.71	0.52
Q8IYB3	SRRM1_S748	Serine and arginine repetitive matrix 1		0.45	
P15336-5	ATF2_T51	Activating transcription factor 2		0.43	
P15336-5	ATF2_T53	Activating transcription factor 2		0.43	
Q6Y7W6	GIGYF2_S26	GRB10 interacting GYF protein 2		0.43	0.53
Q96GN5-4	CDCA7L_S105	Cell division cycle associated 7 like		0.42	
Q04637-8	EIF4G1_S1188	Eukaryotic translation initiation factor 4 gamma 1		0.42	0.39
Q8WYA6	CTNNBL1_S545	Catenin beta like 1		0.41	
Q9NWK9	ZNHIT6_S25	Zinc finger HIT-type containing 6		0.41	
Q03188	CENPC_S316	Centromere protein C		0.38	
Q7L4I2	RSRC2_S32	Arginine and serine rich coiled-coil 2		0.35	
Q7L4I2	RSRC2_S30	Arginine and serine rich coiled-coil 2		0.34	
P49736	MCM2_S139	Minichromosome maintenance complex component 2		0.32	
Q14687-3	GSE1_S753	Gse1 coiled-coil protein		0.31	
Q14687-3	GSE1_S755	Activating transcription factor 2		0.31	
Q5T200	ZC3H13_S325	Zinc finger CCCH-type containing13		0.31	
Q9Y2W1	THRAP3_S928	Thyroid hormone receptor associated protein 3		−0.40	
Q9NQ55-2	PPAN_S359	Peter pan homolog		−0.41	
Q13523	PRPF4B_S87	Pre-mRNA processing factor 4B		−0.41	

Q13523	PRPF4B_S93	Pre-mRNA processing factor 4B		−0.41	
O95685	PPP1R3D_S74	Protein phosphatase 1 regulatory subunit 3D		−0.44	
O95685	PPP1R3D_S78	Protein phosphatase 1 regulatory subunit 3D		−0.44	
Q8WXI9	GATAD2B_T120	GATA zinc finger domain containing 2B		−0.45	−0.42
Q14155-1	ARHGEF7_S340	Rho guanine nucleotide exchange factor 7		−0.50	
Q15052	ARHGEF6_S488	Rho guanine nucleotide exchange factor 6		−0.50	
Q07866-7	KLC1_S547	Kinesin light chain 1		−0.52	
Q12802	AKAP13_S1559	A-kinase anchor protein 13		−0.55	
P57682	KLF3_S92	Kruppel- ike factor 3		−0.57	
P21333-2	FLNA_S2144	Filamin A		−0.58	
P04049	RAF1_S43	Raf-1 proto-oncogene serine and threonine kinase		−0.61	
P13796	LCP1_S257	Lymphocyte cytosolic protein 1		−0.63	
P33241	LSP1_S204	Lymphocyte specific protein 1		−0.79	
Q92835-2	INPP5D_S1038	Inositol polyphosphophate-5-phosphatase D		−0.80	
P43405	SYK_S297	Spleen associated tysone kinase		−0.80	
Q7Z5R6	APBB1IP_S526	Amyloid beta precursor protein-binding family B member 1 interacting protein		−0.85	
O75995	SASH3_Y316	SAM and SH3 domain containing 3		−0.86	
B2RUZ4	SMIM1_S27	Small integral membrane protein 1		−0.91	
I3L1I5	SPATA22_S94	Spermatogenesis associated 22		−0.98	
Q13576	IQGAP2_S16	IQ motif containing GTPase activating protein 2		−1.17	
P26038	EZR_T558	Ezrin		−2.06	
Q9UQ35	SRRM2_S1329	Serine and arginine repetitive matrix 2			0.94
Q9UEY8-2	ADD3_S649	Adducin 3			0.94
Q8IWS0	PHF6_S145	PHD finger protein 6			0.52
Q86WB0	ZC3HC1_S335	Zinc finger C3HC-type containing 1			0.52
Q9BUT9	MCRIP2_S82	MAPK regulated corepressor interacting protein 2			0.45
O75976	CPD_T1368	Carboxypeptidase D			0.42
O75976	CPD_T1370	Carboxypeptidase D			0.42
Q86WB0	ZC3HC1_S407	Zinc finger C3HC-type containing 1			0.40
Q9UQ35	SRRM2_S1320	Serine and arginine repetitive matrix 2			0.36
P32519	ELF1_S168	E74 like ETS transcription factor 1			−0.30
Q96ST2	IWS1_S398	Interacts with SUPT6H, CTD assembly factor 1			−0.32
Q96ST2	IWS1_S400	Interacts with SUPT6H, CTD assembly factor 1			−0.32
P42166	TMPO_S159	Thymopoietin			−0.53
